# Impact of SARS-CoV-2 Pandemic on Psychosocial Burden and Job Satisfaction of Long-Term Care Nurses in Poland

**DOI:** 10.3390/ijerph19063555

**Published:** 2022-03-17

**Authors:** Katarzyna Tomaszewska, Bożena Majchrowicz, Marek Delong

**Affiliations:** 1 Department of Nursing, Institute of Health Protection, The Bronisław Markiewicz State Higher School of Technology and Economics, 37-500 Jarosław, Poland; 2 Department of Nursing, Institute of Social and Health Sciences, East European State Higher School, 37-700 Przemyśl, Poland; bozenam4@op.pl; 3 Department of Management, University of Technology, 35-959 Rzeszów, Poland; m.delong@prz.edu.pl

**Keywords:** nurse, long-term care, psychosocial burden, SARS-CoV-2, psychosocial risk scale

## Abstract

Psychosocial consequences of the coronavirus pandemic are severe for health care workers due to their higher levels of exposure. Nurses often experience tremendous psychological pressure as a result of their workload in a high-risk environment. The purpose of this study was to determine the impact of the SARS-CoV-2 pandemic on the psychosocial burden and job satisfaction of nurses employed in long-term care. One hundred thirty-eight nurses employed in long-term care participated in the study. The respondents were 96.4% female and 3.6% male. The mean age of the respondents was 53.99 (standard deviation—4.01). The study was conducted between February and June 2021. The research tool was a standardized psychosocial risk scale questionnaire, which is a scientifically validated diagnostic tool with high reliability and accuracy coefficients. The primary tests used during the statistical analyses were non-parametric Mann–Whitney U (for two samples) and Kruskal–Wallis (for more than two samples) tests for assessing differences. During these analyses, in addition to standard statistical significance, appropriate *p*-values were calculated using the Monte Carlo method. Correlations between ordinal or quantitative variables were made using Spearman’s rho coefficient. The results obtained allow us to conclude that the respondents rated the characteristics present in the workplace that constitute psychosocial risks at an average level. Emotional commitment and continuance-type commitment to the respondents’ job position were also at a medium level. Respondents’ self-rated ability to work for nurses employed in long-term care during the SARS-CoV-2 pandemic and commitment to patient care was high at 4.0 and 4.18, with a maximum of 5 points.

## 1. Introduction

COVID-19 is a disease caused by a coronavirus infection (SARS-CoV-2), with the first cases diagnosed in 2019 in China. Due to the rapid spread of the disease, the World Health Organization (WHO) declared it a pandemic in March 2020. According to Johns Hopkins University, as of 17 August 2020, there were 21,901,102 laboratory-confirmed cases of coronavirus, including 774,299 deaths worldwide. The International Council of Nurses (ICN) reported that more than 600 nurses worldwide had died from COVID-19 by 3 June 2020. [1]. As of today (December 2021), the SARS-CoV-2 coronavirus outbreak continues, with the daily number of cases increasing, and the fourth wave of the pandemic refuses to subside. On New Year’s Day, 1 January 2022, the Ministry of Health reported another 12,032 cases of coronavirus infection in the country; 505 have died. Poland is approaching the alarming number of 100,000 deaths from the epidemic (currently 97,559) [2].

The COVID-19 pandemic, like any health crisis, negatively affects the well-being of individuals, thereby affecting groups and entire societies. In a pandemic, people experience a range of negative emotions such as feelings of danger, fear, uncertainty, frustration or anger; they tend to be sad, lonely and confused. These emotions lead to suffering and destroy well-being, satisfaction and satisfaction with life. The emotions not only reduce the quality of life but also lead to mental health problems. The most important source of anxiety in a pandemic is, of course, the disease itself and its consequences: we fear for our own health and that of our loved ones, and these fears are often accompanied by a fear of death. Anxiety can also involve isolation, distancing, prohibition of movement, obligation to wear masks and often limits cognitive and social functioning [3].

Statistics maintained by the Supreme Council of Nurses and Midwives (NRPiP) show that there were more than 230,000 active nurses in Poland in 2021. There were about 5.1 nurses per 1000 inhabitants, and their average age was 52.59, which is not a satisfactory result when compared with the data of OECD countries (Organization for Economic Cooperation and Development). In the next 4 years, about 25% of all nurses will reach retirement age and will be entitled to a pension. Currently, in Poland, “the retirement age is 60 years for women and 65 years for men”. Considering the fact that in a pandemic, people experience a range of negative emotions such as a sense of threat, fear, uncertainty, frustration or anger, which leads to suffering, destroys well-being, satisfaction and satisfaction with life, reduces its quality and leads to mental health problems, the number of active nurses may still decrease [4].

The COVID-19 pandemic has placed a significant burden on the health care system. WHO has called for action to reduce its impact on the mental and physical health of health workers. Previous experience with viral outbreaks demonstrates that those delivering health services directly in inpatient care, as well as in-home care, are at increased risk of infection, depression and stress symptoms [5]. In 1984, the International Labor Organization defined psychosocial risks as a kind of interaction between job content, work organization, management systems, conditions and competences, needs and individual characteristics of a worker. The International Labor Organization’s approach is the source of the most current and widely accepted definition of psychosocial occupational hazards, indicating that “psychosocial occupational hazards are those aspects of work organization and management, together with their social and environmental context, that have the potential to cause psychological, social or physical harm” [6].

Since the beginning of the pandemic, nurses have been exposed to a high emotional burden due to longer and closer contact with patients with COVID-19 and at the same time more exposure to the infection than other health care workers, which negatively affects their mental health, occurrence of anxiety and depression. Findings from multiple authors show that a higher mental burden was more prevalent during the first period of the pandemic (spring 2020), decreasing during the second period (fall 2020). This decrease in the second period may be due to the fact that at the beginning of the pandemic (the “first wave”), there was a lot of confusion, lack of information, lack of training of these professionals, lack of PPE, high number of infections in both groups and lack of diagnostic testing. In the second period, the training improved, as well as the level of knowledge of nurses about the COVID-19 virus [7].

Nurses often face tremendous psychological pressure as a result of overwhelming workloads, 12 h on-call schedules (also at night) and working in high-risk environments [8], not only in hospitals or clinics, but also in long-term care [9]. Given the increasing demands on those employed in health care, it is crucial to understand and address the psychosocial burden on staff. These efforts must seek to alleviate major sources of anxiety among health care workers during the SARS-CoV-2 pandemic. Some of these concerns include access to personal protective equipment, balancing one’s own mental and physical health with patient care, fear of exposing family members to the virus, supporting other family members, fear of developing symptoms and increased work demands [10,11,12]. Moral damage or “psychological distress” resulting from actions or lack thereof has been highlighted during the pandemic as a particular threat to health care providers. As Greenberg et al. eloquently articulated, health care personnel “will be the heroes of the day, but we will need them for tomorrow.” Health care workers must receive the support necessary to reach their full potential over the long term [13]. Risks associated with working with patients during the SARS-CoV-2 pandemic, fear of infection, unpredictability of events, feelings of helplessness and anxiety about performing existing job duties are just a few of the elements that nurses currently face while working [14]. Therefore, the mental health of nurses working with COVID-19 infected patients must be monitored and maintained during the outbreak [15]. The services provided will only be of high quality if the work environment provides nurses with the right conditions to support them [16].

According to recent reports, nurses are the health care workers who had the most psychological problems as a result of the COVID-19 outbreak. Although the initial impact on their mental health is evident, at some point, they seem to have adapted to the “new normal” [17]. However, the reporting of mental health problems among nurses during the COVID-19 pandemic worldwide is very low. According to WHO, the COVID-19 pandemic may have both long- and short-term effects on mental health; therefore, it is necessary to address the impact of COVID-19 on nurses’ mental health [18].

In our study, we aimed to assess the impact of the SARS-CoV-2 pandemic on the psychosocial burden and job satisfaction of nurses working in long-term care, as well as to determine to what extent the fulfillment of professional tasks and shift work affects job satisfaction and whether the stress associated with contact with patients infected with coronavirus may cause the desire to change jobs.

## 2. Materials and Methods

### 2.1. Research Design

In the present study, a survey was conducted among nurses employed in inpatient and residential long-term care in Podkarpackie Voivodeship (Poland) who were providing work during the survey. Its aim was to assess the impact of the SARS-CoV-2 pandemic on the psychosocial burden and job satisfaction of nurses working in long-term care. The survey was conducted between February and June 2021.

### 2.2. Methods

The study of psychosocial risks at work and their consequences in the study group was carried out using the Psychosocial Risk Scale (SRP), which is a scientifically validated diagnostic tool and is characterized by high coefficients of reliability and accuracy. The SRP makes it possible to assess the presence of work characteristics that can be potentially threatening and to evaluate their level of stressfulness. It also takes into account such aspects of employees’ functioning as absenteeism at work, frequency of accidents at work, health condition and ability to work, satisfaction with seven aspects of work, involvement in work and willingness to change work. The scale consists of four parts. Part A includes demographic data; Part B contains questions related to health and occupational functioning; Part C consists of 50 statements related to job characteristics that represent potential psychosocial risks. These characteristics are grouped into three main factors: job content, job context and interpersonal pathologies, which in turn consist of bundles of questions belonging to the nine psychosocial dimensions of the work environment listed in Cox’s theory (job content, temporal frame of work, workload, control, organizational culture and functions, interpersonal relationships, role in organization/responsibility, career development, work-home relationship). Part D is a set of statements related to job characteristics specific to occupations in a given economic sector, with reference to our study in the health care sector. The psychometric properties of Parts B and C of the Psychosocial Risk Scale were determined by surveying 7623 respondents. The value of α-Cronbach’s internal consistency coefficient for the whole scale was 0.94 [19].

### 2.3. Participants

The study group consisted of 138 nurses employed in long-term inpatient and in-home care. Inclusion criteria were employment in long-term care with the same provider for at least 1 year and occupation as a nurse. The exclusion criteria were lack of consent to participate in the study and employment of less than 1 year in long-term care. The questionnaires were handed out to the nurses and, after completion, were personally collected by the authors of the study, who clarified any doubts on an ongoing basis.

### 2.4. Statistical Analysis

The primary tests used during the statistical analyses were the non-parametric Mann–Whitney U test (for 2 samples) and Kruskal–Wallis test (for more than 2 samples) to assess differences. During these analyses, in addition to standard statistical significance, the corresponding *p*-values were also calculated using the Monte Carlo method. This is indicated by (b) next to the significance result for the Mann–Whitney U test and by (c) for the *p*-value result of the Kruskal–Wallis test. Correlations between ordinal or quantitative variables were made using Spearman’s rho coefficient, which indicates the intensity of the relationship and its direction—positive or negative. The resulting value ranges from −1 to 1, with (−1) indicating a perfect negative correlation and (1) a perfect positive correlation. The Monte Carlo method, in most cases, is based on a sample of 10,000 tables with the starting number of random number generator 2,000,000. The analysis was performed using the IBM SPSS 26.0 package (IBM, New York City, NY, USA) with the Exact Tests module. All relationships were considered statistically significant when *p* ≤ 0.05.

### 2.5. Ethical Procedures

The participation of nurses in the study was voluntary and anonymous. The study was conducted in accordance with the ethical standards of the Declaration of Helsinki (64th WmA General Assembly, Fortaleza, Brazil, October 2013) and in accordance with Polish legal regulations. The study was approved by the Bioethics Committee (KB/PWSW/1/2022).

## 3. Results

The survey was conducted among 138 randomly selected nurses employed in long-term inpatient and residential care in Podkarpackie voivodeship. All respondents correctly completed the survey questionnaire; the rate of correct answers was *N* = 138. The characteristics of the study group are shown in Table 1.

The data in Table 2 represent the results of Part A of the questionnaire, which includes questions on demographic data such as gender, age, education, job title, length of service, form of employment, shift work performed and number of persons in the household.

### Questionnaire Results

Results obtained during statistical analysis were related to mean scores for the prevalence of health-care-specific psychosocial risks (Part D of the Psychosocial Risk Scale). In our own study, 96% of the respondents believed that the workplace in which the services were provided fully provided employees with personal protective equipment and staff shortages resulted in increased hours of work. Despite the increased workload, the number of sick leave did not increase, averaged 0.87, and the number of days absent from work in the past year was ±8.46. Self-assessment of ability to work during the SARS-CoV-2 pandemic and commitment to work among the surveyed nurses was high at 4.0 and 4.18, with a maximum of 5 points. The overall mean of the psychosocial risk characteristics present in the workplace was found to be low (0.42 ± 0.17). The emotional involvement of the respondents was at a mean level of 2.14 with a maximum of 4 points, as well as the involvement of the “persistence” type in the occupation, which averaged 2.32 with a maximum of 4 points.

Table 3 shows those job characteristics that are health-care-specific, belonging to the job content category and industry-specific included in Part D of the Social Risk Scale.

Among nurses who cared for coronavirus-positive patients, statistically significant negative correlations were found between the mean of occurring workplace characteristics that constitute psychosocial hazards and global job satisfaction and job satisfaction scores on a scale of 1 to 5. The significant strength of the relationship indicates that a higher mean of psychosocial hazards present in the workplace is associated with lower global job satisfaction. On the other hand, a slightly less pronounced correlation coefficient value indicates that respondents experiencing higher psychosocial risks have lower job satisfaction scores on a scale of 1 to 5 (Table 4).

Comparing the number of people and number of children in the household with global job satisfaction showed negative statistically significant correlations, which were characterized by weak strengths of association. A higher number of people and higher number of children in the household were associated with lower global job satisfaction due to stress and risk of COVID-19 virus transmission to the home environment. Considering age, total job tenure and tenure in the current job, there were no statistically significant correlations with global job satisfaction, the average of job characteristics present in the workplace that constitute psychosocial risks and satisfaction ratings with the current job on a scale of 1 to 5. When analyzing global job satisfaction during the SARS-CoV-2 pandemic, the average of job characteristics present in the workplace that constitute psychosocial risks, and satisfaction ratings on a scale of 1 to 5, only the results of the latter variable varied significantly by gender as evidenced by the *p*-values of the Mann–Whitney U test. It is women who, in comparison to men, rated the level of job satisfaction higher. The analysis of the Mann–Whitney U test showed that the level of global job satisfaction, the average of job characteristics constituting psychosocial risks at work and the evaluation of satisfaction with the current job on a scale from 1 to 5 were not statistically significant, as differentiated by the level of education. The results of global job satisfaction, the mean of job characteristics constituting psychosocial hazards in the workplace and the rating of satisfaction with current job performance on a scale of 1 to 5 were not statistically significant, as differentiated by the form of employment, as evidenced by the *p*-values of the Mann–Whitney U test.

Shift work significantly differentiated global job satisfaction, the average level of psychosocial hazards present in the workplace and satisfaction with current job on a scale from 1 to 5, as evidenced by the Mann–Whitney U test results. Respondents who work shifts, compared to others, had lower global job satisfaction, experienced higher levels of psychosocial hazards and rated their job satisfaction lower (Table 5).

## 4. Discussion

Our study investigated the impact of the SARS-CoV-2 pandemic on the psychosocial burden and job satisfaction of nurses employed in long-term care in Poland. Surprisingly, in the obtained results of the statistical analysis of the survey questionnaire, nurses showed less work-related stress than we expected. Of the nurses surveyed, 87.0% stated that their job position had procedures for dealing with patients during a pandemic. For 71.7% of the respondents, responsibility for human health and life or readiness to react quickly (86.2% of the respondents) were not a problem. In our study, according to the respondents, the overall mean of the psychosocial risk factors at work was 0.42 ± 0.17. Emotional commitment of the respondents was at the mean level of 2.14 with a maximum of 4 points, as well as commitment at the occupied workstation, which was at the mean level of 2.32 with a maximum of 4 points. Studies by other authors have confirmed the relationship between psychosocial risk factors [20], which included high job demands, low job autonomy, low control, high effort-reward imbalance, interpersonal conflict, low social support, low trust and employee anxiety and stress [21].

According to Mlokosiewicz, more than a quarter of Polish employees experienced stress at work every day, with almost one-third of employees (32%) believing that the company was not interested in their psychological well-being. According to the employees’ assessment, stress related to psychosocial hazards ranked first among other hazards (physical, chemical and biological) in the workplace (indicated by up to 72% of respondents) [22]. In the study by Kowalczuk et al., the nurses rated the demands of their jobs high (mean score about 3.5). The ability to control their work and the level of social support were rated at an average level (mean score of 3.01 and 3.06, respectively). Respondents rated satisfaction with life rather high (mean score of 3.62). The scale of desirable changes (3.57 points) that should take place in their work was rated high [23]. Based on the results of the review conducted by Al Thobaity and Alshammari, most of the problems faced by nurses in dealing with patients with COVID-19 could be divided into two types. The first consists of staffing shortages, depression due to anxiety and fear of infection, lack of communication with patients and exhaustion due to long hours without adequate food. The second type includes lack of medical supplies and materials, such as personal protective equipment [24]. In our study, 96% of the respondents believed that the workplace where the services were provided fully provided personal protective equipment to the employees and staff shortages resulted in increased hours of work. Working more hours was moderately correlated with fear and anxiety (*p* ≤ 0.012). In the study by Lin et al., almost half of the students surveyed (49.1%) would give up their choice of nursing as a career. The analysis showed that fear of COVID-19 (β = 0.226, *p* < 0.001) influenced the intention to change the field of study [25]. Alnazly and Hjazeen’s study showed that nurses had moderate levels of anxiety (mean score: 24.34 ± 13.43) and depression (43.8% of the sample) and severe anxiety (73.8%) and stress (45.4%). Nurses who cared for patients who tested positive for coronavirus in 2019 and those who had friends or family members who tested positive had higher levels of anxiety and distress (*p* < 0.001 and *p* = 0.010) [26].

Increasing psychological problems of medical workers, mainly nurses and more often women than men, concern increased levels of anxiety, depression, insomnia, chronic fatigue and stress. They especially fear for their own health and that of their families, bear the burden of emotional contact with patients and are subject to occupational overload due to staff shortages and inadequate personal protective equipment. In a state of mental decompensation, they require reliable information support, stress and tension reduction and rest. In the case of continuous work for many hours, they should be guaranteed a place for solitary rest and relaxation and their daily needs such as food, sleep, protective clothing and contact with family [13]. Cengiz et al. showed that the level of participants’ effort in complying with personal protective equipment (PPE) was very high (more than 84.0%). The analysis showed that only 61.1% of the participants could sleep well and regularly. It was found that 85.0% of the participants followed the quarantine rules, 75.7% paid attention to social distancing while working and 81.7% followed the social distance rule in the places where they ate. It was found that 46.7% of the participants feared that they might be carriers of COVID-19 and 38.9% feared COVID-19 infection [27]. In a study by Sikaras et al., 52.4% of the respondents worked in COVID-19 units, the results of 67.9% and 42.9%, respectively, suggested the occurrence of fatigue and burnout among them and showed a strong positive correlation (*p* < 0.01, r = 0.70) [28]. The findings of Alameddine et al. showed that 67.8% of the nurses were satisfied with their jobs and most of the nurses stated that they were unlikely to quit their jobs in the coming year (76.2%). Nurses’ resilience was directly related to job satisfaction (*p* < 0.05) [29]. Similarly, in our study, women compared to men rated job satisfaction higher. Higher mean psychosocial risks at work are associated with lower overall job satisfaction. Studies by Najder and Potocka have shown that the mere presence of psychological workload in the work environment, even if their presence is not stressful for employees, significantly correlates with health and occupational functioning [30], which has not been confirmed by our study.

Prevention of occupational stress, which is becoming an increasingly common health risk for those working with COVID-19 patients, is a major challenge, mainly for occupational health services. Improving psychosocial working conditions and reducing the stress experienced by workers contributes to maintaining and improving their health, as well as maintaining their ability to work. This is particularly important in a coronavirus pandemic situation. In addition, a friendly environment, as well as the fact that the employer cares about the health of employees, promotes greater work engagement [31].

### Limitations of the Study

Nurses in this study working in long-term care were aware that the patients either tested positive for COVID-19 or were suspected cases. The questionnaire used is a self-report instrument; that is, current psychological well-being influenced the respondents’ assessment of the situation. In addition, due to the nature of the long-term residential and home care services provided, the study had a small sample size and large sex bias, which means that the generalizability of the findings is limited.

## 5. Conclusions

A survey of nurses providing inpatient and residential long-term care services in the Podkarpackie Voivodeship (Poland) provides insight into the impact of the SARS-CoV-2 pandemic on their psychosocial burden and job satisfaction. Self-assessment of work ability of surveyed nurses employed in long-term care during the SARS-CoV-2 pandemic and involvement in patient care was high at 4.0 and 4.18 with a maximum of 5 points. The results indicate that the overall average psychosocial distress in the workplace was below health norms. The emotional involvement of the respondents was at an average level, and the higher the level of psychosocial risks present, the lower the global job satisfaction of the respondents.

The results of the presented study allow us to assume that since the onset of the COVID-19 pandemic, the nurses interviewed, along with the gained experience and greater knowledge, have changed their relationships with patients infected with the coronavirus and the level of fear and anxiety was reduced, which positively affects the quality of nursing care. Additionally, introduced vaccinations and mutation of the virus (currently OMICRON) influence severity, in many cases, being asymptomatic COVID-19 among nurses and patients covered by long-term care.

## Figures and Tables

**Table 1:** Characteristics of the study group of nurses.

Variable		Respondents (*N* = 138)	
Sex	Female	133	96.4%
	Male	5	3.6%
Position	Nurse	138	100.0%
Education	Secondary and post-secondary education	47	34.1%
	Higher	91	65.9%
Type of contract	Employment contract for an indefinite period	133	96.4%
	Contract of mandate/contract for specific work	5	3.6%
Shift work	No	50	36.2%
	Yes	88	63.8%

**Table 2:** Results of Part A of the Psychosocial Risk Scale questionnaire.

Group	Mean *	Median **	Standard Deviation	Minimum	Maximum
Age (years)	53.99	54.00	4.01	34	64
Total length of service	33.75	32.50	27.95	4	352
Length of service in current position	23.52	25.00	10.59	1	43
Number of persons in the household	3.22	3.00	1.54	0	10
Number of children in the household	1.03	1.00	1.15	0	5

* Mean value obtained in individual data in 138 respondents. ** The value of a feature in an ordered series, above and below which there are an equal number of observations.

**Table 3:** The most common characteristics of nurse work in health care (*N* = 138).

Characteristics of Nurse Work	Mean	Median	Standard Deviation	Minimum	Maximum
My work requires the use of modern technology.	1.87	2.00	0.538	1	4
My work requires readiness to respond quickly most of the time.	2.05	2.00	0.424	1	4
My work requires adherence to strictly defined procedures.	2.12	2.00	0.499	1	4
My work is often controlled (internal and external audits, visits, quality control, etc.).	1.99	2.00	0.656	1	4
There is an employee evaluation system at my work.	2.03	2.00	0.672	1	4
My work requires constant improvement of qualifications.	1.97	2.00	0.672	1	4
At work I am exposed to psychological aggression from patients (shouting, verbal abuse, blackmail, threats, etc.).	1.40	1.00	0.788	1	4
I am required to be available at work.	2.01	2.00	0.598	1	4
I work under particularly difficult physical conditions.	1.79	1.00	1.000	1	4
At work I am exposed to physical aggression from patients (beating, pushing, pulling, using dangerous tools).	1.08	1.00	0.402	1	4
My work requires a lot of physical effort.	2.22	2.00	1.025	1	4
My work is connected with responsibility for health and life of other people.	2.32	2.00	0.683	1	4
My work requires close cooperation in a team.	1.98	2.00	0.330	1	3

**Table 4:** Correlation of sociodemographic data with Psychosocial Risk Scale scores (*N* = 138).

Spearman’s Rho		Age	Total Length of Service	Length of Service in Current Position	Number of Persons in the Household	Number of Children in the Household
Global job satisfaction (total satisfaction 7–28)	Correlation coefficient	0.078	0.119	−0.014	−0.173 *	−0.226 **
	Relevance (two-sided)	0.360	0.163	0.874	0.042	0.008
Average of workplace characteristics that constitute psychosocial risks (0–1)	Correlation coefficient	−0.145	−0.106	0.038	0.081	0.091
	Relevance (two-sided)	0.089	0.217	0.661	0.344	0.287
Satisfaction with current job (1–5)	Correlation coefficient	0.042	0.050	−0.135	−0.076	−0.134
	Relevance (two-sided)	0.624	0.558	0.114	0.377	0.117

* Correlation significant at the 0.05 level (two-sided). ** Correlation significant at the 0.01 level (two-sided).

**Table 5:** Job characteristics constituting psychosocial risks and satisfaction levels of shift nurses.

Shift Work		Global Job Satisfaction (Total Satisfaction 7–28)	Average of Workplace Characteristics That Constitute Psychosocial Risks (0–1)	Satisfaction with Current Job (1–5)
No	Average	21.24	0.35	3.92
	Median	21.00	0.34	4.00
	Average rank	83.07	52.65	84.74
	*N*	50	50	50
	Standard deviation	3.73	0.13	0.80
Yes	Average	19.52	0.46	3.41
	Median	20.00	0.46	3.50
	Average rank	61.79	79.07	60.84
	*N*	88	88	88
	Standard deviation	3.17	0.18	0.83
Total	Average	20.14	0.42	3.59
	Median	20.00	0.39	4.00
	*N*	138	138	138
	Standard deviation	3.47	0.17	0.85
Mann–Whitney U		1521.500	1357.500	1438.000
*p*		0.003	0.000	0.000
*p* (Monte Carlo)		0.002	0.000	0.000
